# Ovule and seed production patterns in relation to flower size variations in actinomorphic and zygomorphic flower species

**DOI:** 10.1093/aobpla/plz061

**Published:** 2019-09-23

**Authors:** Jun Mochizuki, Tomoyuki Itagaki, Yuta Aoyagi Blue, Masaya Ito, Satoki Sakai

**Affiliations:** Graduate School of Life Sciences, Tohoku University, Aoba, Sendai, Japan

**Keywords:** Angiosperms, ovule number variation, ovule size variation, seed size variation, selection through pollinators

## Abstract

Zygomorphic flower species tend to show lower flower size variation than actinomorphic flower species. Have these differences also brought an association in ovule and seed production that has arisen due to natural selection in these species? Flowers were collected from 29 actinomorphic and 20 zygomorphic flower species, and fruits were collected from 21 actinomorphic and 14 zygomorphic flower species in Miyagi and Aomori prefectures, in Japan. The coefficient of variations (CVs) of flower sizes, mean ovule sizes of flowers, ovule numbers of flowers and mean seed sizes of fruits were calculated. The CV of flower sizes was marginally different between the floral symmetry types; tending to be lower in the zygomorphic flower species than in the actinomorphic flower species. The CVs of mean ovule sizes and ovule numbers of flowers increased with increase in the CV of flower sizes in the actinomorphic flower species but not in the zygomorphic flower species. Mean ovule number of flowers tends to increase with increase in mean flower size in the actinomorphic flower species but not in the zygomorphic flower species. The degrees in variations in ovule size and number of flowers were influenced by the interaction of floral symmetry type and flower size variation, suggesting that floral symmetry also has brought an evolutionary association in ovule production by flowers.

## Teaser

Actinomorphy and zygomorphy are widely observed floral symmetries. We found that variations in mean ovule size and ovule numbers in flowers increased with an increase in variation of flower size in the actinomorphic flower species but not in the zygomorphic flower species. Thus, the degree of variation in ovule size and number of flowers was influenced by the interaction of floral symmetry type and flower size variation, suggesting that floral symmetry also has an evolutionary association with ovule production by flowers.

## Introduction

Animal-pollinated flowers show several patterns of floral symmetry and two types of floral symmetry, actinomorphy (radial symmetry) with several symmetry planes, and zygomorphy (bilateral symmetry) with one symmetry plane, are typical (Neal *et al.* 1998; Endress 2001). Actinomorphy is ancestral, and zygomorphy has evolved independently throughout angiosperms (Stebbins 1974; Coen *et al.* 1995; Endress 2011; Reyes *et al.* 2016; Sauquet *et al.* 2017). Despite such parallel evolution, respective species groups with actinomorphic and zygomorphic flowers show functionally similar pollination patterns, although exceptions also exist; zygomorphic flowers are often visited by a narrower range of pollinator groups than actinomorphic flowers (Richards 1997; Fenster *et al.* 2004; Gong and Huang 2009, 2011). This may be because zygomorphic flowers have complex structures to which bees can respond, whereas actinomorphic flowers have simple structures that many pollinator groups can visit (Coen *et al.* 1995; Richards 1997; Wolfe and Krstolic 1999). Thus, selection through pollinators may differ between actinomorphic and zygomorphic flowers.

This difference leads to difference in flower size variation within species among actinomorphic and zygomorphic groups; zygomorphic flower species tend to show lower flower size variation than actinomorphic flower species (Wolfe and Krstolic 1999; Ushimaru *et al.* 2004; Herrera *et al.* 2008; van Kleunen *et al.* 2008; Gong and Huang 2009; Nikkeshi *et al.* 2015). This may be because, in zygomorphic flower species, accuracy of physical fit between flowers and pollinators is necessary, resulting in low flower size variation (several relevant hypotheses are summarized by Nikkeshi *et al.* 2015).

Selection for flower size and its constancy may also affect ovule and seed production. This may occur in two ways. First, the size of a flower affects the number of pollinator visits to the flower (Young and Stanton 1990; Vaughton and Ramsey 1998; Totland 2001; Celedon-Neghme *et al.* 2007; Teixido and Valladares 2014; Teixido *et al.* 2016; Lebel *et al.* 2018) and physical fit between flowers and pollinators (Schemske and Horvitz 1989; van Kleunen *et al.* 2008; Nikkeshi *et al.* 2015). Then, quantity of pollen a flower is expected to receive may depend on its flower size. This expected quantity of pollen received may affect the number of ovules produced by the flower, and further may affect individual ovule sizes and resulting seed production. Second, it is possible that flowers with different sizes receive different amounts of resources for ovule and seed production (Ashman and Hitchens 2000; Itagaki and Sakai 2006), and in addition, differences in the quantity of pollen the flower is expected to receive may enhance different resource allocation. Thus, ovule and seed production could be also differentiated between actinomorphic and zygomorphic flowers. For example, larger flowers might produce more ovules and seeds in actinomorphic flower species, whereas such a pattern might not be observed in zygomorphic flower species, because larger flowers are expected to receive more pollen in actinomorphic flowers but this trend may weak in zygomorphic flowers.

In ovule and seed production, size and number of ovules and seeds produced are very important elements, and are particularly affected by quantity of pollen the flower is expected to receive and amount of resources available. Many studies have reported variation in seed size among flowers within species (e.g. Michaels *et al.* 1988; Lalonde and Roitberg 1989; McGinley 1989; Kang *et al.* 1992; Sakai and Sakai 1995; Sakai and Harada 2001), and in general, plants with large resource status produce large seeds (Roff 1992; Venable 1992). Several non-mutually exclusive hypotheses have been proposed; e.g. the number of pollen grains received saturates with increase in flower size, resulting in an increase in seed size with flower size rather than in seed number (Sakai and Sakai 1995). However, Greenway and Harder (2007) have shown that variation in ovule size also exists; it is about twice as much as variation in ovule numbers of flowers within species. Thus, it is also possible that different selections on flower size between actinomorphic and zygomorphic flowers lead to different selection on ovule and seed size variations between these groups. However, no previous study has examined this topic despite sizes and numbers of ovules and seeds being very important factors affecting plant reproduction.

The objective of this study was to examine whether ovule and seed production is affected by floral symmetry using 49 naturally growing plant species (Table 1). In particular, we asked the following questions:

**Table 1.** List of the 49 species studied. *A: actinomorphy; Z: zygomorphy.

Species	Family	Floral symmetry*	Sampling site	Sampling habitat
*Chloranthus serratus*	Chloranthaceae	Z	Hakkoda	Forest floor
*Trientalis europaea*	Primulaceae	A	Hakkoda	Forest edge
*Lysimachia vulgaris* var*. davurica*	Primulaceae	A	Hakkoda	Forest edge
*Schizocodon soldanelloides* var*. soldanelloides*	Diapensiaceae	A	Hakkoda	Forest edge
*Oxalis acetosella* var*. longicapsula*	Oxalidaceae	A	Hakkoda	Forest edge
*Chamerion angustifolium*	Onagraceae	Z	Hakkoda	grassland
*Geranium thunbergii*	Geraniaceae	A	Hakkoda	grassland
*Drosera rotundifolia*	Droseraceae	A	Hakkoda	Marshland
*Tiarella polyphylla*	Saxifragaceae	Z	Hakkoda	Forest floor
*Swertia japonica*	Gentianaceae	A	Hakkoda	Wayside
*Gentiana triflora* var. *japonica*	Gentianaceae	A	Hakkoda	Forest edge
*Lotus corniculatus* var*. japonicus*	Fabaceae	Z	Hakkoda	Wayside
*Melilotus officinalis* subsp*. albus*	Fabaceae	Z	Hakkoda	Wayside
*Impatiens noli-tangere*	Balsaminaceae	Z	Hakkoda	Forest edge
*Impatiens textorii*	Balsaminaceae	Z	Hakkoda	Forest edge
*Sanguisorba tenuifolia*	Rosaceae	A	Hakkoda	Forest edge
*Agrimonia pilosa* var. *viscidula*	Rosaceae	A	Hakkoda	Forest edge
*Nephrophyllidium crista-galli* subsp*. japonicum*	Menyanthaceae	A	Hakkoda	Marshland
*Lobelia sessilifolia*	Campanulaceae	Z	Hakkoda	Marshland
*Eupatorium glehnii*	Asteraceae	A	Hakkoda	Forest edge
*Salvia nipponica*	Lamiaceae	Z	Aobayama	Forest floor
*Isodon trichocarpus*	Lamiaceae	Z	Hakkoda	Forest edge
*Mimulus sessilifolius*	Phrymaceae	Z	Hakkoda	Wayside
*Viola mandshurica*	Violaceae	Z	Aobayama	Grassland
*Viola rostrata*	Violaceae	Z	Aobayama	Wayside
*Viola grypoceras* var*. grypoceras*	Violaceae	Z	Izumigatake	Forest edge
*Hypericum erectum*	Hypericaceae	A	Hakkoda	grassland
*Corydalis fumariifolia* subsp*. Azurea*	Papaveraceae	Z	Izumigatake	Forest floor
*Corydalis incise*	Papaveraceae	Z	Aobayama	Forest edge
*Aquilegia buergeriana* var*. oxysepala*	Ranunculaceae	A	Hakkoda	Forest edge
*Anemone pseudoaltaica*	Ranunculaceae	A	Izumigatake	Forest floor
*Aconitum japonicum* subsp*. Subcuneatum*	Ranunculaceae	Z	Hakkoda	Forest edge
*Ranunculus silerifolius* var*. Silerifolius*	Ranunculaceae	A	Izumigatake	Forest edge
*Commelina communis*	Commelinaceae	Z	Izumigatake	Forest edge
*Paris tetraphylla*	Melanthiaceae	A	Hakkoda	Forest floor
*Helonias orientalis*	Melanthiaceae	A	Hakkoda	Marshland
*Disporum sessile*	Colchicaceae	A	Hakkoda	Forest floor
*Disporum smilacinum*	Colchicaceae	A	Aobayama	Forest floor
*Erythronium japonicum*	Liliaceae	A	Aobayama	Forest floor
*Tricyrtis affinis*	Liliaceae	A	Aobayama	Forest floor
*Cardiocrinum cordatum*	Liliaceae	Z	Aobayama	Forest floor
*Hemerocallis dumortieri* var*. esculenta*	Xanthorrhoeaceae	Z	Aobayama	Forest edge
*Allium tuberosum*	Amaryllidaceae	A	Hakkoda	Wayside
*Sisyrinchium rosulatum*	Iridaceae	A	Aobayama	Wayside
*Iris gracilipes*	Iridaceae	Z	Aobayama	Forest edge
*Maianthemum japonicum*	Asparagaceae	A	Hakkoda	Forest floor
*Polygonatum odoratum* var*. maximowiczii*	Asparagaceae	A	Hakkoda	Forest edge
*Maianthemum dilatatum*	Asparagaceae	A	Hakkoda	Forest edge
*Hosta sieboldii* var*. sieboldii* f. *spathulata*	Asparagaceae	Z	Aobayama	Forest floor

(1) Is there difference in the coefficient of variations (CVs) in flower sizes, mean ovule sizes, ovule numbers of flowers and mean seed sizes between the actinomorphic and zygomorphic flower species?
(2) Is there difference in mean flower sizes, mean ovule sizes, mean ovule numbers of flowers and mean seed sizes between the actinomorphic and zygomorphic flower species?
(3) Does the CV of flower sizes affect the CV of mean ovule sizes of flowers, ovule numbers of flowers and mean seed sizes of fruits?
(4) Do mean flower sizes of species affect mean ovule sizes and mean ovule numbers of flowers in the species?

## Materials and Methods

### Study sites and species studied

This study was conducted in natural habitats in Aobayama (N 38.258, E 140.837) and Izumigatake (N 38.247, E 140.4252), Miyagi prefecture, and Hakkoda (N 40.396, E 140.526), Aomori prefecture, in the northern region of Honshu, Japan, during 2015–17. Details regarding the species studied and their sampling sites are provided in Table 1. We studied 49 animal-pollinated, herbaceous species with hermaphroditic flowers from 29 families including 29 actinomorphic and 20 zygomorphic flower species. We avoided examining plant species that produce very small ovules, such as the Orchidaceae species, because individual sizes of these ovules are hard to measure. We sampled plants from a single population for each species, selecting populations with >60 plants. All sample plants in each species were growing in a similar environmental condition.

### Measurements of flowers and ovules

We collected fresh flowers to avoid possible changes in flower sizes with their ages and in ovule sizes by fertilization. For each species, we collected 1–2 fresh flowers from each of about 30 plants. For each plant from which two flowers were sampled, we collected all samples simultaneously so that there was little difference in flower ages among the samples. However, for the species whose individual flowers produce single ovules, i.e. *Agrimonia pilosa* var. *viscidul*, *Sanguisorba tenuifolia*, *Melilotus officinalis* and *Eupatorium glehnii*, we sampled 3–5 flowers from each plant. We avoided selecting plants growing close (within ~10 m) to each other to minimize the chances of studying genetically identical plants. The samples were collected during April 2015 to September 2017.

We determined the size of each flower sampled using one of the following three measurements for each species **[see****Supporting Information—Table S1****]**: petal length, petal mass and petal area. For measurement of petal length, we measured the length of the longest petal of each sample flower in the field using a digital venire caliper (Mitutoyo Corp., Kawasaki, Japan) to the nearest 0.01 mm. We carefully flattened petals to exactly measure their lengths. For measurements of petal mass, the samples collected (all petals of each sample) were dried in an 80 °C oven for 3 days, and then weighed. For measurements of petal area, the largest fresh petal of each sample flower (all of the species whose petal areas were measured were schizopetalous flower species) was scanned using a GT-S630 scanner (Seiko Epson, Tokyo Japan), and its area was measured using ImageJ imaging software (Schneider *et al.* 2012). Because dimensions differ between length (1-dimension), area (2-dimensions) and mass (3-dimensions), and these differences possibly affect CV, we used area^1/2^ and mass^1/3^ values for petal area and mass, respectively, for further calculations. We then calculated the CVs of flower sizes within species without distinguishing plants. Here, we failed to measure the flower size of *Hosta sieboldii* var*. sieboldii* f. *spathulata* and *Viola rostrate*, and hence 47 species were analysed.

We also determined the mean size of the ovules of each flower sampled using one of the following two measurements for each species **[see****Supporting Information—Table S1****]**: ovule area and ovule mass. Before the measurements, we removed ovules from the ovaries and counted the number of ovules of each sample flower under a stereomicroscope. We sampled all ovules of each flower for the most species, but for the species whose individual flowers produce hundreds of ovules, i.e. *Helonias orientali*s, *Chamerion angustifolium*, *Mimulus sessilifolius*, *Gentiana triflora* var*. japonica* and *Schizocodon soldanelloides* var*. soldanelloides*, we randomly sampled 10 ovules for each flower. For each sample flower, the total area or the total dry mass of all ovules counted were measured using the same way for the petal measurements. For the measurements of ovule area, ovules were carefully placed between clear plates so that their largest surfaces facing upward. Then, the mean ovule size (mean area or mean dry mass) of the sample ovules was calculated. We also used mass^2/3^ values for mean ovule mass to remove the effects of different dimensions between area and mass. We calculated the CVs of mean ovule sizes of flowers within species without distinguishing plants.

For the species in which all ovules of each flower were measured, we also counted the number of ovules of each flower, and calculated the CVs of ovule numbers of flowers within species without distinguishing plants.

### Measurements of seeds

Regarding seed production, we concentrate on their sizes, but not on their numbers, because seed number should be highly variable due to pollination event and the effects of flower size variation may be hard to detect.

We collected mature fruits from 35 species, including 21 actinomorphic and 14 zygomorphic flower species, of the above 49 species (Table 1) during May 2015 to October 2017. For the species whose individual fruits produce many seeds, we collected one mature fruit from each of about 30 plants whose flowers had not been sampled. Exceptionally, several fruits were sampled from each plant of *Nephrophyllidium japonicum* because we could collect only a part of seeds produced in each sample fruit. We also sampled 3–5 fruits from each plant for the species whose individual fruits produce single seeds. We avoided selecting plants growing close to each other to minimize the chances of studying genetically identical plants.

We determined the mean seed size (mean area or weight) of each fruit for each species using the same way as that for the ovule measurements. In these measurements, mean seed sizes were calculated using all seeds sampled for the most species, but in several species, mean seed sizes were calculated using 5 or 10 seeds from each fruit **[see****Supporting Information—Table S1****]**. We calculated the CVs of mean seed sizes of fruits within species without distinguishing plants, using mean^2/3^ values for mean mass.

### Statistical analysis

To apply phylogenetically controlled analyses, we obtained the phylogenetic relationships of the species studied (provided in the **Supporting Information**) using the *Phylomatic* online program (version 3) (Webb and Donoghue 2005, http://www.phylodiversity.net/phylomatic). For the ‘*megatree*’, from which phylogenetic information was extracted, we used a tree (R20120829 Phylomatic tree for plants; https://github.com/camwebb/tree-of-trees) derived from Angiosperm Phylogeny Group III (The Angiosperm Phylogeny Group 2009) implemented in the *Phylomatic* program. After obtaining the tree topology, branch lengths were estimated as previously described (Grafen 1989) using the *compute.brlen* function in the *ape* package of R (Paradis *et al.* 2004).

We examined the differences in the CVs of flower sizes, mean ovule sizes of flowers, ovule numbers of flowers and mean seed sizes of fruits and the differences in mean flower sizes, mean ovule sizes, mean ovule numbers of flowers and mean seed sizes between the floral symmetry types (all data are provided in Supplementary Data). Phylogenetic ANOVAs were used for these analyses using aov.phylo function in the *geiger* package of R (Garland *et al.* 1993). In each of these analyses, the floral symmetry type was the explanatory variable and one of the CV, size, and number of the traits examined was the response variable. The number of simulations were 10 000 for each analysis. For the CV of ovule numbers, we excluded species that produce fixed numbers of ovules in flowers (ovule number CV = 0) because fixed production might have evolved due to certain factors, such as morphological constraints, that are unrelated to flower size variation. For the analysis of the difference in mean flower sizes, we concentrated on the species whose petal lengths were measured. This was because species with different flower measurements cannot be included in the same analysis and petal lengths were measured in most species. We examined the differences in ovule and seed sizes using either area or weight, where species having both ovule data were included in both analyses.

We also examined the dependences of the CVs of mean ovule sizes of flowers, ovule numbers of flowers and mean seed sizes of fruits on the CV of flower sizes, and the dependences of mean ovule sizes and mean ovule numbers of flowers on mean flower sizes. Phylogenetic linear regressions were used for these analyses using phylom in the *phylom* package of R (Ho and Ane 2014). In each of these analyses, the CV of flower sizes or mean flower sizes was the explanatory variable and one of the CV, size, and number of the traits examined was the response variable. The analyses were conducted for the actinomorphic flower species and for the zygomorphic flower species separately because our interests are on the dependences within each floral symmetry type. In each analysis, the phylogenetic model for the covariance was the Brownian motion with measurement errors, including environmental variability and sampling error on the species mean, and the number of independent bootstrap replicates was 100. All available species were used for the dependences on the CV of flower sizes, but for the dependences on mean flower sizes, we concentrated on the species whose petal lengths were measured. We analysed the dependences of ovule area and mean ovule number on flower size, but did not analyse the dependences of ovule weight and seed size (seed weight or area) because only a few data were available in each of the floral symmetry types.

All data analyses were conducted using R 3.4.2.

## Results

### CVs of ovule and seed sizes

The CVs of mean ovule sizes of flowers were similar to the CVs of mean seed sizes of fruits; the former ranged from 0.059 to 0.617, with the mean CV of 0.236, and the latter ranged from 0.072 to 0.449 with the mean CV of 0.179.

### Differences in flower, ovule and seed traits between the floral symmetry types

**Figure 1.**
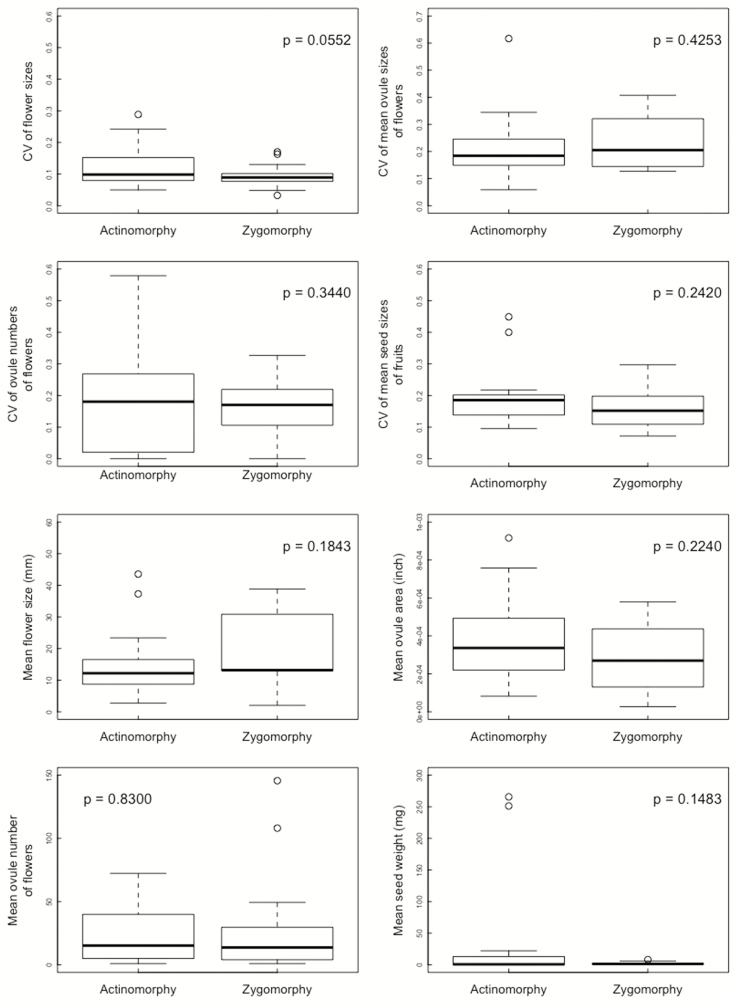
The CVs of flower sizes, mean ovule sizes of flowers, ovule numbers of flowers and mean seed sizes of fruits, and mean flower sizes (petal length), mean ovule sizes (area), mean ovule numbers of flowers and mean seed sizes (weight) in the actinomorphic and zygomorphic flower species.

The CV of flower sizes was marginally different between the floral symmetry types (Table 2; Fig. 1); it tended to be lower in the zygomorphic flower species than in the actinomorphic flower species. There were no significant differences between the floral symmetry types in the CVs of mean ovule sizes of flowers, ovule numbers of flowers and mean seed sizes of fruits (Table 2; Fig. 1).

**Table 2.** Results of the phylogenetic ANOVAs for the difference in CVs of flower sizes, mean ovule sizes of flowers, ovule numbers of flowers and mean seed sizes of fruits and the differences in mean flower sizes, mean ovule sizes, mean ovule numbers of flowers and mean seed sizes between the actinomorphic flower species and the zygomorphic flower species. The analyses were conducted for each response variable separately.

Response variable	Number of species analysed	Estimate	SE	*t-*value	*P-*value
Flower size CV	48	−0.0275	0.0140	−1.9670	0.0552
Ovule size CV	49	0.0233	0.0290	0.8040	0.4253
Ovule number CV (CV = 0 excluded)	34	−0.0327	0.0340	−0.9610	0.3440
Seed size CV	35	−0.0320	0.0269	−1.1930	0.2420
Mean flower size (mm)	31	8.1590	5.9980	1.3600	0.1843
Mean ovule area (inch)	42	−0.00007629	0.00006183	−1.2340	0.2240
Mean ovule weight (mg)	14	0.0076	0.0059	1.2980	0.2190
Mean ovule numbers of flowers	44	1.9920	9.2190	0.2160	0.8300
Mean seed area (inch)	15	−0.0017	0.0016	−1.0720	0.3034
Seed weight (mg)	24	−43.1400	28.7900	−1.4980	0.1483

There was no significant difference in mean flower sizes, mean ovule sizes, mean ovule numbers of flowers and mean seed sizes between the floral symmetry types (Table 2).

### Dependence on the CV of flower sizes

**Figure 2.**
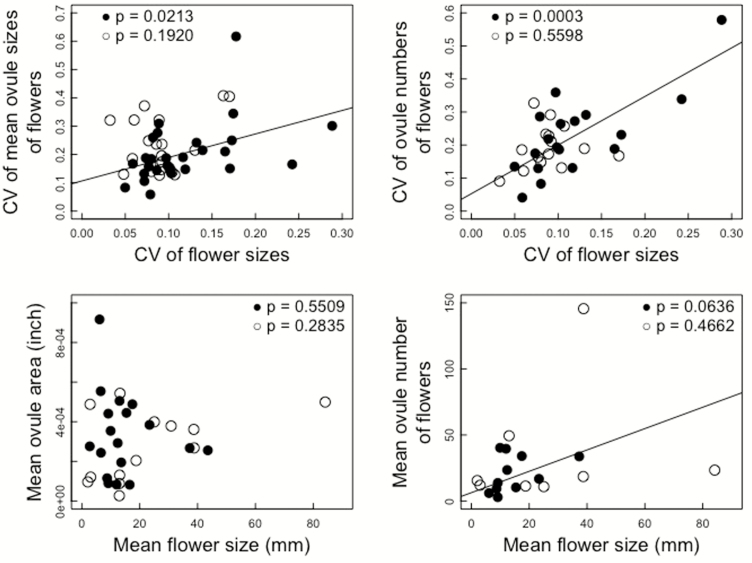
Dependences of the CVs of mean ovule sizes of flowers and ovule numbers of flowers on the CV of flower sizes, and dependences of mean ovule sizes (area) and mean ovule numbers of flowers on mean flower sizes (petal length). Closed circles: actinomorphic flower species; open circles: zygomorphic flower species. The regression lines are provided for significant or marginally significant dependences, and all are for the actinomorphic flower species.

The CV of flower sizes ranged from 0.033 to 0.289 among the species studied. The CVs of mean ovule sizes increased with increase in the CV of flower sizes in the actinomorphic flower species but this was not observed in the zygomorphic flower species (Table 3; Fig. 2). The CVs of ovule numbers of flowers also significantly increased with increase in the CV of flower sizes in the actinomorphic flower species but this was not observed in the zygomorphic flower species (Table 3; Fig. 2). The CV of mean seed sizes of fruits did not significantly depend on the CV of flower sizes in both flower groups (Table 3).

**Table 3.** Results of the phylogenetic linear regression analyses for the dependences of the CVs of mean ovule sizes of flowers, ovule numbers of flowers and mean seed size of fruits on the CV of flower sizes and for the dependences of mean ovule sizes (area) and mean ovule numbers of flowers on mean flower sizes (petal length). The analyses were conducted for the actinomorphic flower species and for the zygomorphic flower species separately, and within each floral symmetry type, the analyses were conducted for each response variable separately.

Response variable	Number of species analysed	Estimate	SE	*t-*value	*P-*value
Actinomorphic flower species					
Ovule size CV	27	0.8296	0.3376	2.4577	0.0213
Ovule number CV (CV = 0 excluded)	18	1.4716	0.3204	4.5930	0.0003
Seed size CV	19	0.0830	0.3602	0.2306	0.8204
Ovule area (inch)	17	−0.000003004	0.000004923	−0.6102	0.5509
Ovule number	15	0.8082	0.3987	2.0274	0.0636
Zygomorphic flower species					
Ovule size CV	20	0.8548	0.6319	1.3527	0.1920
Ovule number CV (CV = 0 excluded)	14	0.3397	0.5663	0.5999	0.5598
Seed size CV	15	−0.1277	0.3616	−0.3530	0.7297
Ovule area (inch)	13	0.000002231	0.000001978	1.1276	0.2835
Ovule number	11	0.4265	0.5606	0.7609	0.4662

Mean ovule number of flowers tended to increase with increase in mean flower size in the actinomorphic flower species (marginally significant) but this was not observed in the zygomorphic flower species (Table 3; Fig. 2). The other traits did not show significant dependence on mean flower size in both floral symmetry types (Table 2).

## Discussion

The CV of flower sizes was marginally, significantly lower in the species with zygomorphic flowers than in the species with actinomorphic flowers. This result is consistent with previous studies on flower size variations in these flower groups (Wolfe and Krstolic 1999; Ushimaru *et al.* 2004; Herrera *et al.* 2008; van Kleunen *et al.* 2008; Gong and Huang 2009; Nikkeshi *et al.* 2015). Thus, selection also favours stable flower size in the present zygomorphic flower species.

The observed CVs of mean ovule sizes of flowers (they ranged from 0.059 to 0.617 with the mean CV of 0.236) for the 49 species studied were similar to those CVs reported in a previous study (Greenway and Harder 2007), in which the CV ranged from 0.153 to 0.663 with the mean CV of 0.356. Thus, both studies showed that the degrees of variation in ovule size considerably differ among species. Also, the CVs of mean ovule sizes of flowers were similar to the CVs of mean seed sizes of fruits observed in this study. Thus, variations in ovule size seem to be high, and there may be some ecological factors selecting ovule size variation, as in seed size variation.

Ovule size variation observed in the present study attributes to the factors relating to the conditions of parents and/or flowers containing ovules rather than to the factors relating to the differences in the environmental conditions among populations and among flowering seasons. This is because those conditions do not differ among sample flowers of the same species since we sampled ovules in certain short periods from single populations for all species studied. Hence, this suggests that the effects of the conditions of parents and/or flowers on ovule size variation should be great.

Also, ovule size variation should not be due to the developmental plasticity because of the following three reasons. (i) We sampled plants from a single population for each species, and sample plants were growing in similar environmental conditions. Hence, plants would have not shown developmental plasticity in ovule development responding to the differences in the growing environments. (ii) We collected fresh flowers to avoid possible changes in ovule sizes with their ages. (iii) For each plant from which several flowers were sampled, we collected fresh samples simultaneously so that there was little difference in flower ages among the samples. Thus, in each species, flowers sampled were fresh, young ones growing in similar environmental conditions, and there should be little room for ovules developing to different sizes by developmental plasticity.

It is noteworthy that the CV of mean ovule sizes of flowers and the CV of ovule numbers of flowers increased with the increase in the CV of flower sizes in the actinomorphic flower species but this was not observed in the zygomorphic flower species (Fig. 2). This suggests that ovule production is correlated with flower size variation in the actinomorphic flower species but not in the zygomorphic flower species.

The observed result in the actinomorphic flower species might be due to the flower size-dependent variation in pollinator visits. In general, frequency of pollinator visits increases with increase in flower size (Young and Stanton 1990; Vaughton and Ramsey 1998; Totland 2001; Celedon-Neghme *et al.* 2007; Teixido and Valladares 2014; Teixido *et al.* 2016; Lebel *et al.* 2018). Hence, it is likely that the number of ovules in a flower depends on its flower size because the expected quantity of pollen the flower will receive increases with increase in its flower size, in addition to a likely factor that the amount of resources available to the flower also depends on its size. Moreover, if increase in pollinator visits to flowers of the species considered diminishes with flower size, the ovule size may also increase with increase in flower size (Sakai and Sakai 1995). This is because, due to the diminishing pollinator visits, increase in ovule number becomes less advantageous with increase in flower size. Then, large flowers, which should have large amounts of resources for ovule production, may invest their resources for the increase in ovule size rather than for the increase in ovule number. Thus, this could be a factor for ovule size variation among flowers within species.

In contrast, in the zygomorphic flower species, stabilizing selection on ovule size and number is not affected by degree of stabilizing selection on flower size, suggesting that stable quantity of pollen is expected to be received irrespective of individual flower sizes. This may be possibly due to these flowers using specific reliable pollinators (Coen *et al.* 1995; Richards 1997; Wolfe and Krstolic 1999), and physical fit between flowers and pollinators, rather than flower size, is important. Here, in the species whose flower size CVs are relatively large, physical fit might be less necessary and stabilizing selection on flower size might be relatively weak. Nevertheless, ovule size and number are also rather stable and hence use of reliable pollinators might also result in receiving a stable amount of pollen even in these species.

Such difference may be also the case for the among-species difference in flower size; mean ovule number of flowers tended to increase with increase in mean flower size in the actinomorphic flower species but this was not observed in the zygomorphic flower species (Table 3; Fig. 2). Thus, the expected quantity of pollen a flower will receive increases with increase in flower size in actinomorphic species, and hence large flowers produce greater numbers of ovules. In contrast, in the zygomorphic flower species, flower size might not affect the expected quantity of pollen received. Thus, pollinator-mediated selection might also affect ovule and flower production even among-species level.

This study showed that the degrees in the variations in ovule size and number were influenced by the interaction of floral symmetry type and flower size variation. Such differences have not been previously found, and this finding may bring new insight to studies of the evolution of ovule and seed production in relation to floral symmetry. In future studies, detailed analyses on the effects of floral symmetry and flower size on pollination will be useful.

## Supporting Information

The following additional information is available in the online version of this article—

**Table S1.** Species examined.

**Supplementary Data.** MochizukiAoBSI.csv

plz061_suppl_Supplementary_Table_S1

plz061_suppl_Supplementary_Data

plz061_suppl_Supplementary_Legends

## Sources of Funding

This study was supported in part by a Grant-in-Aid from the Japanese Ministry of Education, Science and Culture.

## Contributions by the Authors

J.M., T.I. and S.S. designed the work, J.M., T.I., Y.A.B. and M.I. performed the work. J.M. and S.S. wrote the paper.

## Conflict of Interest

None declared.
