# Supplementary material for: Ovule and seed production patterns in relation to flower size variations in actinomorphic and zygomorphic flower species
Source: AoB Plants. 2019 Sep 23;11(5):plz061. doi: 10.1093/aobpla/plz061 (PMC6804480; doi:10.1093/aobpla/plz061)
Supplement: plz061_suppl_Supplementary_Legends [file plz061_suppl_supplementary_legends.docx]

**Table S1.** Species examined.

**Supplementary data.** MochizukiAoBSI.csv
